# Hydrocephalus, cerebral vasospasm, and delayed cerebral ischemia following non-aneurysmatic spontaneous subarachnoid hemorrhages: an underestimated problem

**DOI:** 10.1007/s10143-022-01919-9

**Published:** 2022-12-22

**Authors:** Christina Wolfert, Christoph J. Maurer, Ansgar Berlis, Hauke Schneider, Kathrin Steininger, Stefan Motov, Philipp Krauss, Björn Sommer, Ehab Shiban

**Affiliations:** 1 Department of Neurosurgery, University Hospital Augsburg, Stenglinstr. 2, 86156 Augsburg, Germany; 2 Department of Neuroradiology, University Hospital Augsburg, Stenglinstr. 2, 86156 Augsburg, Germany; 3 Department of Neurology, University Hospital Augsburg, Stenglinstr. 2, 86156 Augsburg, Germany

**Keywords:** Occult subarachnoid hemorrhage, Negative angiography, Cerebral vasospasm, Delayed cerebral ischemia, Hydrocephalus, Outcome

## Abstract

Non-aneurysmal subarachnoid hemorrhage (NASAH) is rare and mostly benign. However, complications such as cerebral vasospasm (CV), delayed cerebral ischemia (DCI), or post-hemorrhagic hydrocephalus (HC) may worsen the prognosis. The aim of this study was to evaluate the rate of these complications comparing perimesencephalic (PM) and non-perimesencephalic (NPM) SAH. Monocentric, retrospective analysis of patients diagnosed with NASAH from 01/2010 to 01/2021. Diagnosis was set only if vascular pathologies were excluded in at least one digital subtraction angiography, and NASAH was confirmed by cranial computed tomography (cCT) or lumbar puncture (LP). One hundred patients (62 female) with a mean age of 54.9 years (27–84) were identified. Seventy-three percent had a World Federation of Neurological Surgeons (WFNS) grading scale score I, while 9% were WFNS score IV or V at the time of admission. SAH was diagnosed by cCT in 86%, in 14% by lumbar puncture. Twenty-five percent necessitated short-term CSF diversion by extraventricular drainage or lumbar drainage, whereof 7 suffered from long-term HC treated with ventriculoperitoneal shunting (VPS). One patient without a short-term CSF drainage developed long-term HC. Ten percent developed CV, four of whom received intraarterial spasmolysis. Radiological DCI was diagnosed in 2%; none of these correlated with CV. Despite a mortality of 3% occurring solely in NPM SAH, the analyzed complication rate was comparable in both groups. We observed post-hemorrhagic complications in 35% of cases during the first 3 weeks after bleeding, predominantly in patients with NPM SAH. For this reason, close observation and cranial imaging within this time may be indicated not to overlook these complications.

## Introduction

Subarachnoid hemorrhage (SAH), most often caused by ruptured aneurysms (85%), has a high case fatality [1]. Nevertheless, in approximately 10–20% of all cases, there are non-aneurysmal SAH (NASAH), neither being caused by vascular pathologies, nor from trauma [2].

Based on the initial bleeding pattern, NASAH is subdivided into perimesencephalic (PM) and non-perimesencephalic (NPM) [3]. In comparison to aneurysmal SAH, better prognosis of PM and NPM SAH are described. However, the current literature suggests to differentiate between PM and NPM SAH due to fundamental differences in clinical course combined with an elevated complication rate in NPM SAH and the increased risk of long-term morbidity [4, 5].

Therefore, this paper aims to evaluate the performed diagnostics, the clinical course as well as, the complications of patients with PM and NPM SAH non-aneurysmal SAH.

## Material and methods

### Study setting and patient selection

**Fig. 1:**
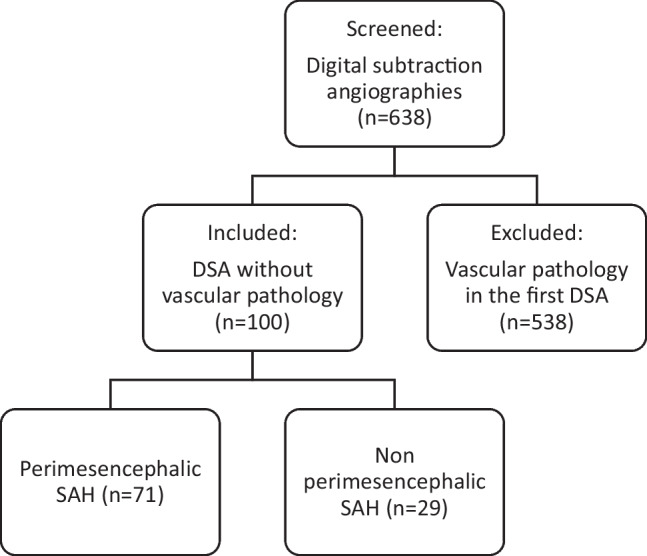
Number of patients screened and included for evaluation

We performed a retrospective, single-center-analysis of patients admitted to the University Hospital Augsburg, Germany, due to spontaneous subarachnoid hemorrhage (SAH), from January 2010 to January 2021 (Fig. 1).

Patients with the history of SAH secondary to a traumatic event were excluded. The remaining SAH patients were selected if at least one digital subtraction angiography (DSA) was performed and excluded underlying vascular pathology (e.g., aneurysm).

Patients aged > 18 years and diagnosed with NASAH by cCT and/or lumbar puncture (LP) were included. Exclusion criteria were (1) intraventricular hemorrhage, (2) mostly convexity hematoma (subdural, epidural hematoma), (3) traumatic SAH, and (4) vascular pathologies in the CTA.

Diagnostic cerebral angiographies were performed with a biplane DSA unit with rotational capabilities (Axiom Artis, Siemens Healthcare, Forchheim, Germany). In general, after femoral access using the Seldinger technique, all four vessels supplying the brain—both internal carotid arteries (ICA) and both vertebral arteries—were catheterized, and nonionic contrast medium was selectively injected. Images were acquired in four planes for each vessel. In case of a suspected or actual vascular pathology on 2D images, 3D rotational images of the corresponding vessel were also acquired. Images were reconstructed in a 512 × 512 matrix. The angiographic rotation data were then transferred to an independent workstation (Leonardo, Siemens Healthcare, Germany) to generate reformatted 3D images. If angiography was unremarkable, selective catheterization of the external carotid artery (ECA) was also performed. Images were usually obtained in two planes to detect or exclude dural arterio-venous fistulas. If the SAH extended to the foramen magnum, images of both subclavian arteries were also acquired to exclude spinal vascular pathologies as a potential but rare cause of SAH.

CCT and CTA were performed using a 64-slice CT scanner (Sensation 64, Siemens Healthcare, Forchheim, Germany) or a second generation dualsource CT scanner (SOMATOM Definition FLASH, Siemens Healthcare, Forchheim, Germany).

Depending on the initial bleeding pattern, patients were divided in the PM and NPM group based on the classification of the study group of van Gijn and Rinkel [6, 7]:

- PM SAH: hemorrhage ventral to the brain stem, mainly in the interpeduncular cistern and/or the ambient and chiasmatic cistern or the horizontal part of the Sylvian cistern.
  NPM SAH: bleeding mostly located in the Sylvian or interhemispheric cistern or the convexity or cCT-negative, but LP positive bleeding (confirmed with a three glasses sample containing not only erythrocytes, but also erythrophages in the cerebrospinal fluid (CSF)).

All data were collected through digital patient records and radiological images. These included patient characteristics, number and type of performed angiograms, as well as the bleeding pattern. Upon admission, the World Federation of Neurological Societies (WFNS) grading scale was applied.

The clinical course and in-hospital complications were documented. Acute hydrocephalus (HC) was defined with the need for an external CSF diversion, either via lumbar drainage (LD) or external ventricular drainage (EVD). While long-term HC was diagnosed in patients’ necessitating a CSF diversion for more than 2 weeks and therefore receiving a ventriculoperitoneal shunt (VPS). CV and DCI have not been defined symptomatically, e.g., as the development of new neurological deficits, but only based on the transcranial Doppler (TCD), cCT, CTA, and MRI findings due to the possibility of missing data.

CV was defined as mean flow velocity > 120 cm/sec or doubling of the mean flow velocity compared to the last TCD [8]. This was further confirmed with CTA and indication for intraarterial spasmolysis was set by the neuroradiologists. DCI was diagnosed as the appearance of new infarction on cCT and MRI. Additionally, rebleeding and in-hospital mortality were documented.

### Ethics

The study was approved by the local ethics committee of Ludwig-Maximilians-University hospital Munich (Reference Nr: 21–1297). Due to the retrospective nature of the study, no informed consent was necessary. The study was performed in accordance with the 1964 Helsinki declaration and its amendments.

### Outcomes

Our primary outcome of interest was the analysis of complications, namely, acute and chronic HC, rebleeding, CV, and DCI comparing PM SAH and NPM SAH. The secondary endpoint was the analysis of periinterventional vessel dissection during DSA.

### Statistical analysis

Continuous data are presented using mean and standard deviation (SD) where appropriate. For categorical data, prevalences using percentages are given. Pearson´s Chi [2] test was used to analyze statistical significance. A *p* value of < 0.05 was considered statistically significant. Data analysis was performed with IBM SPSS v. 25 (IBM Corp, Armonk, USA).

## Results

### Patient population

A total of 100 patients (62 (62%) female and 38 (38%) male) met the inclusion criteria. Mean age was 54.9 years (range: 27–84 years).

**Fig. 2:**
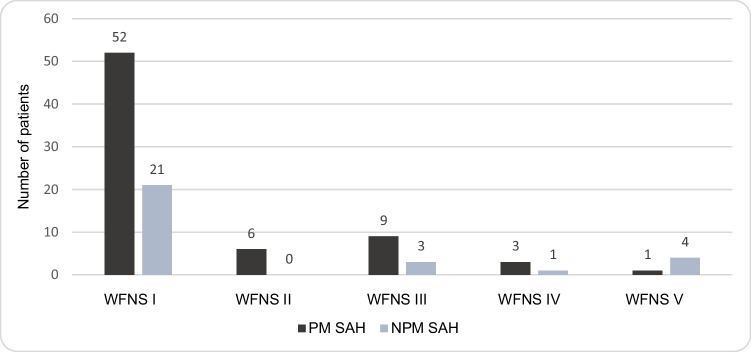
WFNS grade at admission

Most patients were recorded with WFNS score I (PM SAH, 52/71; NPM SAH, 21/29), while in total, 9% was admitted with a WFNS score IV or V (PM SAH: 4/71; NPM SAH: 5/29) as shown in Fig. 2.

Most patients (*n* = 86; PM SAH, 60/71; NPM SAH, 26/29) did not receive antithrombotic medication. Twelve PM SAH patients were taking either platelet inhibition or oral anticoagulation (OAC), and two patients received both OAC and a platelet inhibitor. Demographic data are summarized in Table 1.

**Table 1:** Patient and radiological evaluation characteristics comparing PM and NPM SAH

Baseline characteristics	Non-aneurysmal SAH (*n* = 100)	PM SAH (*n* = 71)	NPM SAH (*n* = 29)	*p* Value
Age (mean; SD)	54.9 ± 12.3	54.9 ± 10.2	54.9 ± 16.6	0.18
Female sex (*n*; %)	62 (62.0)	45 (63.4)	17 (58.6)	0.41
Platelet inhibition (*n*; %)	10 (10)	8 (11.3)	2 (6.9)	0.51
OAK (*n*; %)	6 (6)	4 (5.6)	2 (6.9)	0.65
Number of CTA/MRA (mean; SD)	1.32 ± 0.77	1.34 ± 0.79	1.28 ± 0.75	0.17
Number of DSA (mean, SD)	1.35 ± 0.74	1.35 ± 0.76	1.34 ± 0.72	0.55

*Statistical significance *p* < 0.05

SD, standard deviation; *n* = number of patients; OAK, oral anticoagulation; CTA, computed tomography angiography; MRA, magnetic resonance angiography; DSA, digital subtraction angiography

### Radiological evaluation

The diagnostic confirmation included at least one cCT scan (native/with intravenous iodine-based contrast agent) and one DSA in every patient. Albeit 14 patients (14.0%) did not show any signs of bleeding in the cCT, NASAH was confirmed with LP in these patients.

In total, 135 DSA have been performed, without any statistically significance comparing PM SAH (mean, 1.35; range, 1–4) and NPM SAH (mean, 1.34; range, 1–5). However, follow-up DSA is not routinely performed in our clinic; 26 patients were evaluated with a second DSA. Thereof, 6 underwent multiple (more than two) DSA. Overall, there is a small risk of vessel dissection (2.0%), which was clinically irrelevant. The diagnostics were completed with cCT or MR-angiogram in 89 patients with a total of 132 examinations (PM SAH mean, 1.34; range, 1–4 vs. NPM SAH mean,1.28; range, 0–2).

### Complications

Acute HC treated with EVD (*n* = 24) or LD (*n* = 1) was diagnosed in 25 patients in total (PM SAH, 16/71; NPM-SAH, 9/29; *p* = 0.26). The overall incidence of chronic HC treated with VPS was 8% (PM SAH, 4/71; NPM-SAH, 4/29; *p* = 0.17). One of these patients had a cCT-negative SAH, which was diagnosed by LP, without radiographic or clinical signs of acute HC. The remaining 7 patients (7%) were diagnosed and treated on acute HC prior.

CV was diagnosed in 10 patients (10%), without any significance comparing the initial blood distribution (PM SAH, 6/71; NPM-SAH, 4/29; *p* = 0.47).

However, radiographic findings of DCI solely occurred in patients with PM SAH without correlation to CV and in-hospital mortality (PM SAH 2/71; NPM SAH 0/29; *p* = 0.4). Whereas statistical significance was reached in NPM SAH being associated with an elevated risk of in-hospital mortality (PM SAH, 0/71; NPM SAH, 3/29; *p* = 0.02) and rebleeding (PM SAH, 0/71; NPM SAH, 2/29; *p* = 0.03).

The first patient (82 years, male) died after 5 days due to a massive rebleeding at day 2 with intraventricular and intracerebral hemorrhage.

In another patient (84 years, male), a rebleeding with intraventricular hemorrhage was diagnosed at day four. Two days later, hospital-acquired pneumonia caused respiratory insufficiency leading to reintubation. Due to the presumed patient will, the invasive ventilation was stopped, and the patient died eight days after hospitalization.

The third patient (74 years, male) developed an EVD associated ventriculitis at day five and died 2 weeks later at the palliative ward.

The mean age of these patients was 80 years, being 25.1 years above the average of the patients included. All of them initially suffered from a hemiparesis; where of two were severe and accompanied by reduced vigilance. Further, the in-hospital course was complicated by preclinical aspiration, pneumonia, or ventriculitis.

In conclusion, the rate of the analyzed complications is 35%. Differences in complications are summarized in Table 2.

**Table 2:** Total complications, stratified by initial blood distribution

Complications	All patients (*n* = 100)		PM SAH (*n* = 71)		NPM SAH (*n* = 29)		*p* Value
	*n*	%	*n*	%	*n*	%	
Acute hydrocephalus	25	25.0	16	22.5	9	31.0	0.26
Chronic hydrocephalus (VPS)	8	8.0	4	5.6	4	13.8	0.17
CV	10	10.0	6	8.5	4	13.8	0.47
DCI	2	2.0	2	2.8	-	-	0.44
Intraarterial nimodipine application	4	4.0	2	2.8	2	6.9	0.58
Peri-interventional vessel dissection	2	2.0	1	1.4	1	3.4	0.50
In-hospital mortality	3	3.0	-	-	3	10.3	0.02*
Rebleeding	2	2.0	-	-	2	6.9	0.03*

*Statistical significance

*n* = number of patients; VPS, ventriculoperitoneal shunt; CV, cerebral vasospasm; DCI, delayed cerebral ischemia

## Discussion

### Key findings

In our study, NPM SAH, rebleeding and higher WFNS grades at admission were associated with in-hospital mortality, whereas neither CV, DCI, nor HC altered the in-hospital mortality rate.

Acute HC, treated with EVD or LD was diagnosed in one-fourth of our patients without any significant difference comparing PM and NPM SAH. In their study published in 1992, Rinkel and co-workers suggest that filling of the perimesencephalic cisterns with blood is a necessary factor for the occurrence of acute HC [9]. This was confirmed to be a significant finding in the comparison of PM SAH and diffuse SAH in the investigation by Kang and colleagues, reporting incidences of acute HC to be as high as 25% [10]. However, this findings cannot be reproduced in our study.

A recent study of Achrén and colleagues included 108 spontaneous angiogram-negative SAH patients with 30% of radiological and clinical signs of acute HC [11]. They reported significantly higher HC rates in NPM SAH (*p* = 0.002), being associated with worse clinical outcome, which can be confounded by the presence of IVH in 54% of their study cohort [11].

In comparison, our study excluded patients with IVH, therefore being more accurate in predicting HC being associated with the SAH itself, not altered by IVH.

Another dreaded complication of SAH is CV leading to hypoperfusion of the related brain areas, which may lead to cell death processes and DCI [12].

In aneurysmal and traumatic SAH, CV is diagnosed in approximately 40%, whereas the study of Achrén and co-workers registered CV in 16%, necessitating active treatment in 13% without specifying the treatment itself [11]. Further, a DIC rate of 6% is reported, and both CV and DCI were excluded to statistically significantly correlate with the initial bleeding pattern, which is conclusive to our study results.

Radiological signs of CV were evident in 10% of our patients during the hospitalization, whereof 4 were treated invasively with intraarterial nimodipine. Without being associated to CV, radiological findings of DCI were confirmed in 2%. As a major limitation, our study may underestimate the findings of DCI and CV, since solely radiologically diagnosed DCI and CV were taken into account due to possible data inconsistency.

Despite the occurrence of CV and DCI, mortality being caused by NASAH is scarce. Achrén and colleagues described 1 year overall mortality in five cases, whereof four were related to SAH [11]. However, others published a mortality rate of 10% without identifying the causes of death. Mortality was unrelated to the initial bleeding pattern in this study [13]. However, our findings reveal a 3% rate of in-hospital mortality caused by SAH itself. Mortality was solely documented in NPM SAH and most often caused by early rebleeding. All deceased patients suffered from infections, complicating the in-hospital course. Further, these patients had an advance decision determining that in case of a care-dependent condition life-extending treatments should not be made, potentially leading to an underdiagnosing of a vascular pathology being the source for both, the initial and the rebleed. A specific management to reduce these complications could include early follow-up DSA within 3 days after admission. However, this is no guarantee for the detection of a bleeding source and includes an additional risk of periinterventional vessel dissection.

### Strengths and limitations

The treatment of our patients was not standardized and in-hospital time was short; therefore, some complications can be underestimated. Due to the retrospective nature of this study, CV and DCI could only be evaluated radiologically without any statement about the clinical impact. Additional demographic data such as arterial hypertension and smoking history may vary the clinical course, but could not be evaluated in this study due to missing data in most of the patients. Furthermore, the differing group sizes according to the bleeding pattern make the study subject to type II sampling error, and these results should be interpreted with caution.

Additionally, the initial clinical status can be influenced by the pre-hospital use of sedatives/analgesics, epileptic seizures, and other circumstances which are hard to evaluate in a retrospective setting.

With a comparable amount of PM SAH and NPM SAH, the observed insignificant findings could have become statistically significant. In addition, due to a lack of data, no reference can be made regarding patients’ clinical outcome and possible neurological long-term complications. Therefore, prospective studies are essential to compare the clinical outcome and the radiological findings as well as to evaluate long-term complications.

## Conclusion

Evaluating the complication rate of PM and NPM SAH, it is remarkable that these so-called benign disease harbors a risk of acute HC in 25% and a shunt dependency 8% in our single-center cohort. Because CV (10%) and DCI (2%) were diagnosed only by the radiological evaluation and the clinical status could not be evaluated retrospectively, these complications may be underestimated in our cohort. The analyzed complication rate of 35% was independent of the bleeding pattern. The included mortality rate of 3% was solely observed in NPM SAH, caused by rebleeding in most cases (66.6%). Therefore, close observation and standardized treatment of these patients may be indicated.

## Data Availability

The data that support the findings of this study are not openly available due to reasons of sensitivity and are available from the corresponding author upon reasonable request.
